# Impact of care provider network characteristics on patient outcomes: Usage of social network analysis and a multi-scale community detection

**DOI:** 10.1371/journal.pone.0222016

**Published:** 2019-09-09

**Authors:** Mina Ostovari, Denny Yu

**Affiliations:** 1 Value Institute, Christiana Care Health System, Newark, Delaware, United States of America; 2 School of Industrial Engineering, Purdue University, West Lafayette, Indiana, United States of America; Medical University Graz, AUSTRIA

## Abstract

**Objective:**

We assess healthcare provider collaboration and the impact on patient outcomes using social network analysis, a multi-scale community detection algorithm, and generalized estimating equations.

**Material and methods:**

A longitudinal analysis of health claims data of a large employer over a 3 year period was performed to measure how provider relationships impact patient outcomes. The study cohort included 4,230 patients with 167 providers. Social network analysis with a multi-scale community detection algorithm was used to identify groups of healthcare providers more closely working together. Resulting measures of provider collaboration were: 1) degree, 2) betweenness, and 3) closeness centrality. The three patient outcome measures were 1) emergency department visit, 2) inpatient hospitalization, and 3) unplanned hospitalization. Relationships between provider collaboration and patient outcomes were assessed using generalized estimating equations. General practitioner, family practice, and internal medicine were labeled as primary care. Cardiovascular, endocrinologists, etc. were labeled as specialists, and providers such as radiology and social workers were labeled as others.

**Results:**

Higher connectedness (degree) and higher access (closeness) to other providers in the community were significant for reducing inpatient hospitalization and emergency department visits. Patients of specialists (e.g. cardiovascular) and providers specified as others (e.g. social worker) had higher rate of hospitalization and emergency department visits compared to patients of primary care providers.

**Conclusion:**

Application of social network analysis for developing healthcare provider networks can be leveraged by community detection algorithms and predictive modeling to identify providers’ network characteristics and their impacts on patient outcomes. The proposed framework presents multi-scale measures to assess characteristics of healthcare providers and their impact on patient outcomes. This approach can be used by implementation experts for informed decision-making regarding the design of insurance coverage plans, and wellness promotion programs. Health services researchers can use the study approach for assessment of provider collaboration and impacts on patient outcomes.

## Introduction

Chronic conditions are the leading causes of death and disability, resulting in annual healthcare costs of $3.3 trillion in the United States [1]. Six in ten Americans have at least one chronic condition, while four in ten suffer from two or more [2]. Patients with comorbidities such as hypertension, hyperlipidemia, and diabetes are at higher risk of developing additional chronic conditions [3, 4]. Care of patients with multiple chronic conditions are often complex due to greater healthcare needs that require a larger number of healthcare providers who typically are not co-located at a single health institution [5, 6]. Collaborative approaches for management of chronic conditions are the key to ensure effective healthcare delivery, to prevent health deterioration, and to reduce adverse outcomes for the patients [7, 8], and the objective of this work is to assess how measures of healthcare provider collaboration impact on patient outcomes.

Collaboration encompasses themes such as coordination, cooperation, teams, shared-decision making, and partnerships [9]. Many studies have suggested that these themes impact patient care and outcomes [7, 10, 11]. To measure collaboration, survey tools have been the primary approach [12–14].Studies have shown that collaboration metrics from survey tools are associated with increasing patient safety in hospitals [15] and reducing patient mortality [16]. Although current survey tools capture individual providers perspectives and rapidly scale up to collect information from a large number of participants [17], developing and disseminating surveys can be time consuming and costly, with low response rate [18], and most importantly, survey measures are limited by multiple sources of bias. To better assess impacts of providers collaboration on patient care and outcomes, more objective approaches are needed to study healthcare providers’ relations, team structures, and the associated impacts on patients.

Quantitative data analytics approaches with healthcare data have been rapidly increasing [19–21]. Recent studies with social network analysis (SNA) have shown validity for identifying working relationships between healthcare professionals using large-scale claims data [22, 23]. Barnett et al. used SNA to show that hospitals with physicians whose patients received care from larger number of doctors (showing higher degree centrality) had higher spending compared to other hospitals [24]. Pollack et al. showed that patient whose providers shared higher number of patients (higher care density) had lower rates of adverse events compared to other patients [25]. However, these SNA techniques only examined providers in the overall network (high-level analysis) which may miss behaviors at the care team level. Therefore, more granular assessment of the providers’ team structures is needed.

A modified SNA approach with community detection algorithms [26–28] has been developed to assess physicians’ characteristics in the network and within smaller communities (i.e., sub-dividing a large care network into smaller groups of nodes more densely connected). Using this approach, they showed that patients whose physicians were connected with larger number of physicians had higher spending. Patients in communities with higher proportions of primary care providers had fewer specialist visits and fewer emergency department visits [29]. Hospital networks with higher number of nodes in each community had higher readmission rates, and higher number of nodes in the community was interpreted as physicians having harder time getting the patient information across the community, thereby, negatively impacting patient outcomes [30]. These studies demonstrated that community detection algorithms provide finer-scale measures to predict patient outcomes, however, most algorithms used in these studies for example the Girvan-Newman method [27, 31, 32] suffer from a resolution limit [33] and are unable to detect smaller communities in the network (i.e., more granularity is needed to understand the care team level). Another limitation in previous studies is that application of the community detection techniques has been limited to mainly physicians and hospitals networks [24, 25, 26, 29, 34]. Although constraining networks to focus on the key providers simplifies the model and interpretation, it limits the ability to focus on coordination of chronic care teams that often involves many other care providers. Thus, inclusion of other providers (e.g., pharmacists, nurse practitioner) is necessary to explore flow of information and activities inside the network [35].

The objective of this work is to assess healthcare provider collaboration and the impact on patient outcomes. We propose to address the previously mentioned limitations by integrating multi-scale community detection algorithms with social network analysis to assess the relations at a granular community level. The study hypotheses are as below:

Primary Hypotheses (null): SNA centrality measures (degree, betweenness, and closeness centrality) of the care provider in the community do not impact patient inpatient hospitalization rate (H1), patient unplanned hospitalization rate (H2), and patient emergency department visits rate (H3).

Secondary Hypothesis (H4) (null): Effects of centrality measures (degree, betweenness, and closeness) of the care provider on patient outcomes were consistent over the period from Year 1 (2014) to Year 2 (2015).

## Materials and methods

### Data source

This study was approved by the Purdue University Institutional Review Board (IRB 1511016796). The study data included three years-2014, 2015, 2016- of de-identified claims data from employees (faculty/staff) of a large university in the Midwest. Student population was excluded from this study due to different health service plans. Claims data contained insurance eligibility information, medical and medication services, health services costs, healthcare provider information, and patient outcomes based on the Johns Hopkins Adjusted Clinical Groups version 11.0 [36]. Study data is not publicly accessible due to sensitive patient information; however, interested readers may contact the Regenstrief Center for Healthcare Engineering at Purdue University to request access to data for research purposes.

### Study cohort

The study cohort included patients identified with diabetes, hypertension, and/or hyperlipidemia. Previous work has identified these conditions as most prevalent in the study population [37]. We tracked the cohort healthcare utilization and providers over a three-year period.

### Study design and analysis

The study cohort was identified from the first, second, third, fourth, and fifth diagnosis codes and based on the International Classification of Diseases 9^th^ edition (ICD9). Patients were categorized as a patient with diabetes if one of their diagnosis codes started by 250 [38], hypertensive if one of the codes started with 401 [39], and with hyperlipidemia if one of the codes started with 272 [40]. Health service utilization and associated providers of the patient cohort were identified from the medical file and medication file. Records of patients and their providers were extracted over a three-year period from 2014 to 2016.

### Constructing the network

Previously, we showed that patient sharing among healthcare providers can be used to assess the team structure of patients with diabetes [41, 42]. All providers for the patient cohort were identified for all three years of the study. Separate provider networks were generated for Year 1 (2014) and Year 2 (2015). The network nodes represented healthcare providers and edges represented the patients-sharing relationship. Number of patients shared between healthcare providers was interpreted as the edge weight. Only providers who shared two patients or more were included; previous validation work suggests patient sharing <2 has happened by chance and without significant information sharing values [41].

### Network communities

Granular assessments of the network was performed using a multi-scale community detection algorithm [43] applied to the biggest component of the network [33]. This multi-scale algorithm was previously shown to successfully identify communities of healthcare providers [41] at a better resolution compared to algorithms used in previous work as mentioned in the introduction for identifying smaller health teams from large health datasets. Using the multi-scale algorithm, communities in the network were defined as groups of healthcare providers with denser internal connections compared to their connections with the rest of the network [43]. This approach assigned providers to distinct communities, i.e., each provider appeared once and associated with only one community.

### Assigning patients to providers

Every individual patient was assigned to a “majority source of care provider,” defined as the provider who was responsible for majority of services to the patient during a one-year period based on the number of visits using the Johns Hopkins ACG System [36]. We defined general practitioner, family practice, and internal medicine as “primary care.” Providers such as cardiologists, urologists, and endocrinologists were labeled as “specialists.” Other providers identified (e.g., chiropractors, physical therapists, and diagnostic radiology) were defined as “other”. Patients were assigned to the communities that their majority source of care provider belonged to.

### Network measures of interests

We focused on SNA centrality measures specifically degree, betweenness, and closeness centrality. These measures assessed provider’s connectedness (degree), control over the flow of the information (betweenness), and access (closeness) in the community. The measures are defined as below:

- Degree centrality of the provider showed the direct connections that provider had with other providers in the network [44].
- Betweenness centrality showed the degree to which a provider (node) was between the shortest paths connecting other nodes. Providers with higher betweenness centrality are shown to have more control over the flow of information [24].
- Closeness centrality of the provider in the network showed how close the provider was to other providers in the network. Providers with higher closeness centrality have better access to other providers in the network [45].

We calculated these centrality measures for all providers in the network. The degree, betweenness, and closeness centrality of the majority source of care provider assigned to each patient was identified at 1) the overall network level and 2) at the community level. We first calculated the centrality measures of the majority source of care in the entire network. In the next step, the centrality measures were calculated at the community level where the measures described provider’s position among other providers in the community.

### Patient outcomes of interest

Patient outcomes were defined based on the Johns Hopkins Adjusted Clinical Groups [36] and included: 1) emergency department visit, 2) unplanned hospitalization, and 3) inpatient hospitalization. Unplanned hospitalization refers to all hospitalizations that were not related to a definitely planned or a potentially planned procedure (e.g. cardiovascular, hip replacement) [36]. Emergency visit count considers visits to the emergency department which were not precursor to subsequent hospitalization. The emergency department visits that were followed by a hospitalization were absorbed by that hospitalization [36]. Patient outcomes of interests were extracted for Year 2 (2015) and Year 3 (2016).

### Statistical analysis

Different distributions were tested to find the best distribution fit for the outcome variables and determined the negative binomial [46–48] as the best fit distribution to model the data, and thus, was used in the following outcomes modeling.

To test our hypotheses about the impact of community-level centrality of the care provider on patient outcomes, we used the generalized estimating equations (GEE) models. As GEE models do not make any assumption about the distribution of the data, they provide more robust results compared to other models, e.g., hierarchical models [29]. In addition, GEE allows accounting for the clustering of observations and similarities between patients that belong to the same community (patients were assigned to the community of their majority source of care provider). To account for this clustering, GEE fits marginal regression models with variances adjusted for the clustering [49]. Separate models were generated for each outcome (emergency department visit, inpatient hospitalization, and unplanned hospitalization). The predictors included community and network level centrality (degree, betweenness, and closeness), patient age, sex (male/female), and type of majority source of care providers (i.e. primary care, specialist, or other providers such as social worker). The interactions of the centrality measures with year were also considered to account for the longitudinal analysis. Due to different ranges of the network variables, we standardized all the continuous variables in the model so they all had mean of 0 and standard deviation of 1. The β coefficient of each independent variable can be interpreted as a change in the outcome variable of interest for each standard deviation change in the independent variable, representing the standardized effect size. The GEE model is described in Eq 1.

Model: Negative Binomial Regression

$$g(\mu_{i})=X_{i}^{T}\beta$$ (1)

Where

$$X_{i}^{T}\beta =\beta_{0}+\beta_{1}x_{1}+\beta_{2}x_{2}+\cdots +\beta_{d}x_{d}$$

With link function

g(.)=log(.)

$$\mu_{i}∼Negative\;binomial\;distribution$$

Where i represents the i^th^ subject

The relations between independent and outcomes variables were modeled as follows: network characteristics in Year 1 (2014) were associated with outcomes in Year 2 (2015), and network characteristics in Year 2 were associated with outcome variables in Year 3 (2016). As suggested by previous work, a one year lag accounted for the time needed for provider collaboration to impact outcomes [25, 29]. All analyses were completed using SAS (v 9.4, SAS Inc., Cary, NC) and RStudio (version 0.99.903) with the igraph (version 1.1.2) [50] and devtools (version 1.12.0) [51] packages.

## Results

A total of 19,247 patients were identified in Year 1 (2014) with at least one medical claims. Out of these patients, 4,395 patients were identified with diabetes, hypertension, and/or hyperlipidemia. The cohort received healthcare services from 2,332 providers in Year 1, 2,421 providers in Year 2, and 2,444 providers in Year 3.

### Network-level characteristics

Out of 2,332 providers in Year 1, only 38% (N = 894) shared two patients or more. Thus, the network of Year 1 had 894 nodes (providers) with 8,853 edges. The biggest component of the network had 890 nodes and 8,851 edges. The two other components had each 2 nodes and one edge. As the network only included providers with two patients or more in common, these smaller components were generated as those providers (nodes) only shared one patient with the providers in the bigger component of the network. Focusing on the biggest component of the network, the median degree centrality of the network nodes was 7, the median betweenness centrality was 146.86, and the median closeness centrality was 0.000193.

The network of Year 2 (2015) had 930 nodes with 9,631 edges. The biggest component of this network had 924 nodes and 9,624 edges. Similar to network of Year 1, two smaller components with 4 nodes and 2 nodes were also generated. In the biggest component of the network, the median degree, betweenness, and closeness centrality were 8, 132.94, and 0.000188 respectively.

### Communities-level characteristics

To conduct a more granular assessment of the full SNA network, a multi-scale community detection algorithm [52] was applied on the biggest component of the networks of Year 1 and Year 2 to separate the large network into smaller distinct communities, increasing our ability to understand meaningful and closer collaborations among providers. Nineteen communities were detected for the network of Year 1 with 2 to 193 nodes in each community. Twenty-one communities were detected for the full network of Year 2 with 2 to 197 nodes in each community.

### Analysis of patient outcomes

In Year 1 and Year 2, 212 and 214 unique providers, respectively, were determined as majority source of care providers. Fifty-two percent (N = 86) of these providers were primary care. Forty-three percent (N = 72) were identified as specialists, and only 5% (N = 9) were identified as “other providers”. Patient outcomes from these providers were linked to the provider SNA measures for testing our hypotheses (H1 through 4). There were 4,230 patients extracted; 2,113 females with average age of 55.78±9.53, and 2,117 males with average age of 56.37±10.8. Table 1 describes the outcome variables in the dataset for Year 1 and Year 2.

**Table 1:** Description of the outcome variables.

	Inpatient Hospitalization (%)	Unplanned Hospitalization (%)	Emergency Department (%)
**Year 1**^**1**^	128 out of 4230 (3%)	109 out of 4230 (2.5%)	612 out of 4230 (14%)
**Year 2**^**1**^	136 out of 4230 (3%)	134 out of 4230 (3%)	608 out of 4230 (14%)

^**1**^ Number of patients that had that outcome at least once

To model the relation between provider network measures of collaboration and patient outcomes, generalized estimating equations generated marginal negative binomial models (distribution determined using Vuong’ closeness test) after adjusting the variance structure for patient’s community clustering. Models first used both majority source of care providers’ *network-level* centrality measures (network degree, betweenness, and closeness) and *community-level* centrality measures (community degree, betweenness, and closeness). *Network-level* measures were not statistically significant in any of the outcome variable models. Thus, models in the following results used only *community-level* centrality measures. Table 2 to 4 present the results of the GEE models for unplanned hospitalization, inpatient hospitalization, and the emergency department visit. The estimate of the independent variable is the difference in the logarithm of the outcome variable when independent variable increases by one standard deviation. The exponential of the estimate (Exp (estimate)) is the rate of change of the outcome variable when the independent variable increases by one standard deviation (i.e., back-transformed estimates).

**Table 2:** GEE model results for the unplanned hospitalization outcome. The estimates are based on the standardized and log transformed data.

*GEE Regression Models For Unplanned Hospitalization*					
*Variable*	*Estimate*	*Exp (Estimate)*	*95% Confidence Limits*	*Z-Statistic*	*P-value*
*Intercept*	-4.11	0.02	(-4.47, -3.76)	-22.6	< .0001
*Age*	0.19	1.21	(-0.01,0.40)	1.88	0.06
*Sex (female)*	-0.039	0.96	(-0.19,0.12)	-0.5	0.62
***Provider type (other)***	**0.91**	**2.47**	**(0.35,1.46)**	**3.2**	**0.001**
***Provider type (specialist)***	**0.95**	**2.59**	**(0.53,1.37)**	**4.48**	**< .0001**
*Community betweenness*	0.11	1.12	(-0.09,0.32)	1.08	0.28
*Community degree*	-0.21	0.81	(-0.52,0.09)	-1.35	0.18
***Community closeness***	**-0.24**	**0.78**	**(-0.47,-0.02)**	**-2.12**	**0.03**
***Year (year one)***	**-0.14**	**0.87**	**(-0.26,-0.02)**	**-2.3**	**0.02**
*Community betweenness x year*	0.09	1.10	(-0.18,0.37)	0.7	0.48
*Community degree x year*	-0.18	0.84	(-0.45,0.09)	-1.28	0.2
***Community closeness x year***	**-0.23**	**0.79**	**(-0.46,-0.001)**	**-1.97**	**0.04**

QIC = 2296.65, significant variables are bold

**Table 3:** GEE model results for the inpatient hospitalization outcome. The estimates are based on standardized and transformed data.

*GEE Regression Models for Inpatient Hospitalization*					
*Variable*	*Estimate*	*Exp (Estimate)*	*95% Confidence Limits*	*Z-Statistic*	*P-value*
*Intercept*	-4.45	0.01	(-4.41, -3.68)	-21.82	< .0001
***Age***	**0.24**	**1.27**	**(0.03,0.46)**	**2.25**	**0.024**
*Sex (F)*	-0.05	0.95	(-0.2,0.09)	-0.67	0.50
***Provider type (other)***	**0.91**	**2.49**	**(0.30, 1.53)**	**2.92**	**0.003**
***Provider type (specialist)***	**1.11**	**3.04**	**(0.77,1.45)**	**6.37**	**< .0001**
***Community betweenness***	**0.27**	**1.31**	**(0.03,0.4)**	**2.37**	**0.02**
***Community degree***	**-0.34**	**0.70**	**(-0.68, -0.01)**	**-2.04**	**0.041**
***Community closeness***	**-0.37**	**0.68**	**(-0.65,-0.10)**	**-2.72**	**0.006**
*Year*	-0.07	0.93	(-0.17,0.02)	-1.49	0.35
*Community betweenness x year*	0.09	1.09	(-0.19, 0.38)	0.64	0.52
*Community degree x year*	-0.22	0.79	(-0.51,0.06)	-1.54	0.12
***Community closeness x year***	**-0.36**	**0.7**	**(-0.63,-0.08)**	**-2.58**	**0.010**

QIC = 2412.69, significant variables are bold

**Table 4:** GEE model results for the emergency department visits outcome.

*GEE Regression Models for Emergency Department Visits*					
*Variable*	*Estimate*	*Exp (Estimate)*	*95% Confidence Limits*	*Z-Statistic*	*P-value*
*Intercept*	-4.03	0.02	(-4.40,-3.65)	-21.00	< .0001
*Age*	-0.08	0.92	(-0.22,0.05)	-1.22	0.22
*Sex (F)*	-0.010	0.99	(-0.09,0.07)	-0.24	0.81
***Provider type (other)***	**0.35**	**1.42**	**(0.2,0.52)**	**4.21**	**< .0001**
***Provider type (specialist)***	**0.39**	**1.47**	**(0.14,0.64)**	**3.10**	**0.002**
***Community betweenness***	**0.10**	**1.10**	**(0.02,0.18)**	**2.44**	**0.01**
*Community degree*	-0.11	0.89	(-0.23,0.01)	-1.85	0.06
*Community closeness*	-0.03	0.96	(-0.1,0.025)	-1.15	0.25
*Year*	-0.03	0.97	(-0.08,0.03)	-0.96	0.33
*Community betweenness x year*	-0.02	0.97	(-0.11,0.05)	-0.63	0.53
*Community degree xyear*	0.001	1.00	(-0.09,0.09)	0.03	0.97
*Community closeness x year*	-0.03	0.97	(-0.09,0.04)	-0.85	0.4

QIC = 5648.72, significant variables are bold

The estimates are based on standardized and transformed data.

Table 2 represents the results of the GEE model for unplanned hospitalization outcome. Among control variables, patient age and sex were not significant; however, type of provider (i.e. primacy care, specialists, or other providers) was identified as significant (p-value < 0.05). Patients of providers classified as other provider and specialists had 2.47 and 2.59 times more unplanned hospitalization compared to patients of primary care providers. Highest number of patients with at least one unplanned hospitalization belonged to providers classified as other provider (N = 110 for Year 1 and Year 2) followed by patients of specialists (N = 67 for Year 1 and Year 2) and primary care providers’ patients (N = 56 for Year 1 and Year 2).

**Fig 1:**
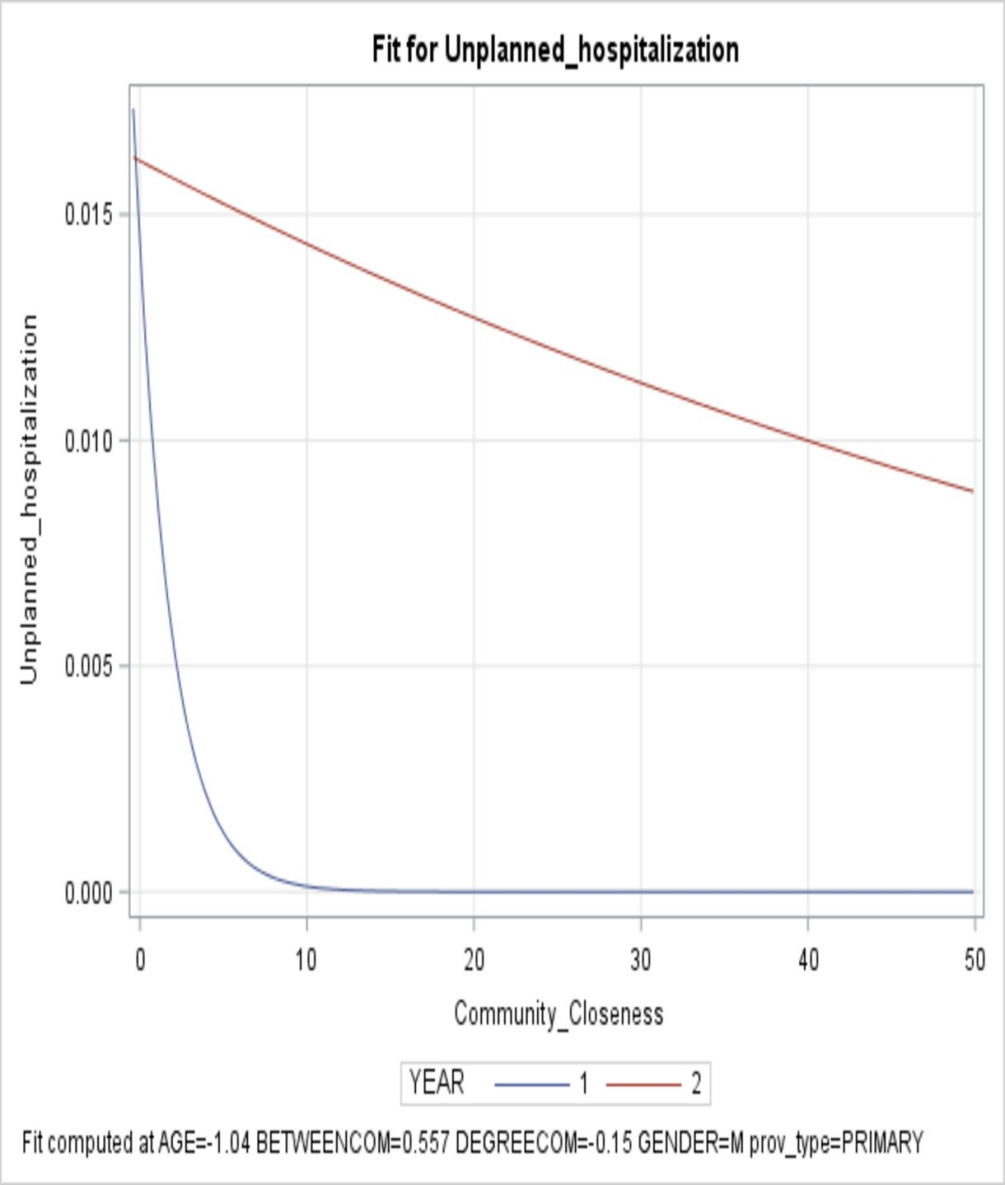
Provider closeness in the community and its effect on unplanned hospitalization in Year 1 vs. Year 2. The x-axis is the closeness of the provider in the community and the y-axis shows the predicted unplanned hospitalization rate.

Community closeness of the majority source of care provider was significant and higher closeness was associated with lower unplanned hospitalization. In addition to significance of closeness, there was an interaction between the provider’s closeness in the community and Year. Specifically, the impact of closeness had different magnitude of effect on unplanned hospitalization in Year 1 vs. Year 2 (Fig 1). Higher provider closeness in the community was associated with lower rate of unplanned hospitalization. When closeness increased by 1 standard deviation of the closeness, the unplanned hospitalization increased by 0.78 for second year and 0.62 for the first year (exp (-0.24–0.23)). Rate of increase smaller than 1 (0.62, 0.78) represent reduction in the rate of unplanned hospitalization.

Table 3 shows the GEE regression results for the inpatient hospitalization outcome. Among the control variables, patient age, and type of majority source of care provider (primary, specialists, or other providers) were significant (p-value<0.05). For 1 standard deviation increase in age, rate of inpatient hospitalization would increase by 1.27. Specialists and other types of majority source of care had 3.04 and 2.49 times inpatient hospitalization among their patients compared to the primary care providers. Highest number of patients with at least 1 inpatient hospitalization belonged to providers classified as other (N = 117 for Year 1 and Year 2) followed by patients of specialists (N = 73 for Year 1 and Year 2) and primary care providers patients (N = 62 for Year 1 and Year 2).

All three centrality measures were statistically significant. Higher provider degree (connectedness) was associated with reduced inpatient hospitalization (negative estimate). As degree increases by 1 standard deviation, the inpatient hospitalization increases by 0.7. Higher provider betweenness (control over flow of information) was associated with increased inpatient hospitalization (positive estimate). As betweenness increases by 1 standard deviation, inpatient hospitalization would increase by 1.31.

**Fig 2:**
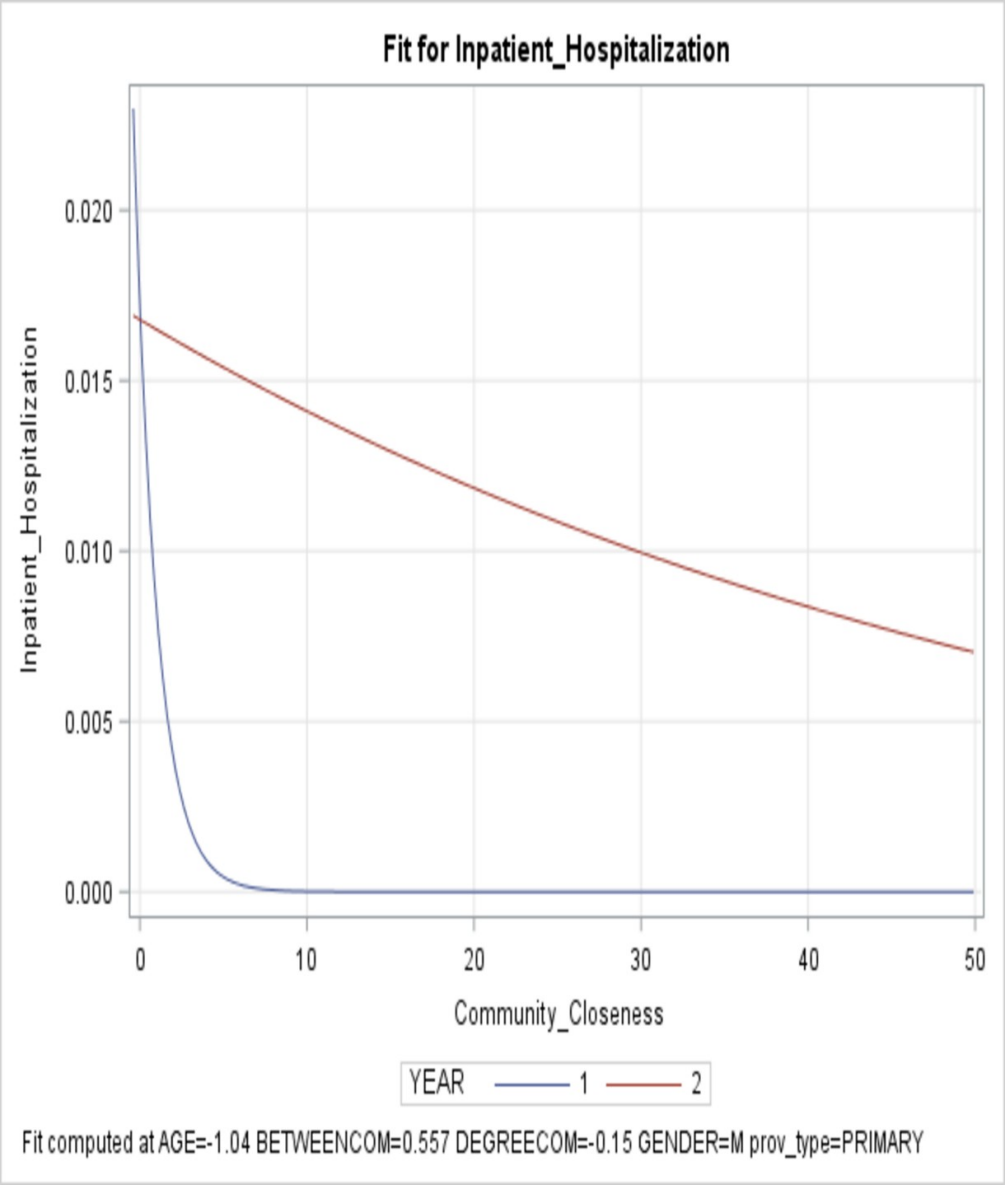
Provider closeness in the community and its effect on inpatient hospitalization in Year 1 vs. Year 2. The x-axis is the closeness of the provider in the community and the y-axis shows the predicted inpatient hospitalization rate.

Similar to unplanned hospitalization, closeness of the care provider in the community was significant and interacted with Year, meaning the effect of closeness on the inpatient hospitalization in both years was significant but the effect differed from Year 1 to Year 2 (Fig 2). Higher closeness in the community was associated with lower inpatient hospitalization rate for both years (Table 3), however, in Year 1 higher closeness reduced patient inpatient hospitalization more than higher closeness in Year 2.

Table 4 shows the result of the GEE model for the emergency department visits outcome. Similar to inpatient hospitalization and unplanned hospitalization, type of majority source of care provider significantly impacted the outcome. Patients with specialists and other types of providers had 1.47 and 1.42 times higher emergency department visits compared to patients of primary care providers as their majority source of care. Highest number of patients with at least one emergency department visit belonged to providers classified as other (N = 449 for Year 1 and Year 2) followed by patients of specialists (N = 378 for Year 1 and Year 2), and primary care providers patients (N = 249 for Year 1 and Year 2).

The only significant community measure for emergency department visits was the majority source of care betweenness in the community. When betweenness increases by 1 standard deviation of the betweenness, the emergency department visit rate increases by 1.1.

## Discussion

This study presented a framework integrating social network analysis, a multi-scale community detection algorithm, and predictive modeling to provide finer-scale assessment of the provider network characteristics and their impact on patient outcomes with health claim datasets. Previous studies that used the SNA application limited their network to mostly physicians, specifically those associated with hospitals [24–26]. Depending on the type of condition, including other providers such as pharmacists, nurse practitioners, and dietitian may be necessary to understand network structure and information sharing patterns [35]. This is especially true for patients with chronic conditions that may have large care teams. In this study, we focused on patients with diabetes, hypertension, and/or hyperlipidemia due to their similar healthcare needs and utilization.

The study population focused on patients with hyperlipidemia, hypertension, and diabetes. As the prevalence of chronic conditions increases, frameworks are needed to monitor and understand on how increasingly complex collaborations impact patient outcomes. Although only three chronic conditions were modeled, the proposed research framework and metrics are not dependent on disease burden and thus likely generalizable to care teams of other illnesses and patient populations.

Based on the results from the GEE models, patients with specialists or providers specified as others as their majority source of care provider had higher rate of emergency department visits, inpatient, and unplanned hospitalization compared to patients with primary care providers as their care managers. Previous studies have suggested that primary care providers are effective team members and typically the center of care for patients [53–55], therefore, they might be better than others at coordinating the team and managing the care process. This may partially explain the observed provider impact on outcomes (Tables 2–4). Another potential explanation for the higher rate of hospitalization and emergency department visits for patients with specialists as their care manager might be due to the more serious health problems that require management by specialist compared to patients with primary care as their care managers. Nevertheless, the study provides evidence that type of provider who manages the care of patients with chronic conditions can help to predict the patient outcomes.

Closeness of the majority source of care provider in the community was a significant predictor for the number of planned and unplanned inpatient hospitalization of the patients; it had a consistently protective effect (negative) on poor outcomes. Higher closeness of a provider in a network represents higher access to other providers [45] and may suggest higher access to information or more familiar coordination among providers. As the care manager of the patient [36], this closeness (access) to other providers in the network and community may be a predictive metric for care quality. For example, lack of information about patient’s previous conditions, hospitalization, and emergency department visits have shown to negatively impact the patient hospitalization [56]. Thus, care managers higher access to other providers may allow them to better coordinate and manage the care process and flow of information among other involved providers to reduce potential negative impacts on patients.

Despite the important implication of provider greater closeness (access) to patient care, the impact on outcomes interacted with Year in the present study. Although this may suggest inconsistency and unreliability of closeness-centrality as a predictor of patient outcomes, we believe this interaction highlights the sensitivity to changes in care collaborations. Provider relationships and network characteristics are impacted by multiple factors outside of the study control variables. Specifically, factors like insurance coverages [25] can disrupt team collaborations. For the present study population, two major changes occurred in insurance coverages. First the Affordable Care Act was implemented on January 1^st^ of Year 1 (2014) which required all individuals to have insurance coverages. Second, a health savings plan was introduced in addition to the previously flexible spending accounts which allowed the insured individual’s unused health funds to roll over to the next year. It is important to note that despite this interaction and difference in the magnitude of the impact, the direction of the closeness effect was consistently protective to poor outcomes.

Based on the regression results, providers’ higher connectedness to others in the community (higher community degree) was associated with lower inpatient hospitalization. A physician higher degree indicates that the provider shares patients with higher number of providers. Previous studies have shown conflicting results about the providers’ degree. According to Barnet et al. higher degree of providers in the network was associated with higher patient cost and utilization of services [24]. In contrast, another study identified that provider’s larger connectedness was associated with fewer adverse outcomes, and larger degree was associated with lower readmission rate after hospitalization for heart failure [57]. The present study showed that higher connectedness lowered risk of adverse events for the studied chronic conditions, which aligns with literature emphasizing the important of care collaboration and ease of dissemination of information which can positively impact the patient outcomes in chronic disease management.

In this study, we identified healthcare providers’ centrality measures at both whole-network level and the more closely working together community-level. Previous studies that assessed relations between provider centrality (degree and betweenness) and patient outcomes explored those characteristics for the entire network [24, 29,57]; however, the whole network centrality measures were not significant predictors for modeling of patient outcomes and only community-level metrics were significant for our dataset. This suggests the algorithm used in this study is able to identify smaller communities in the network that might be a better reflection of the care teams. The finer-scale community measures may provide better reflection of the provider characteristics and its associated impact on patients compared to those measures in the entire network. These more granular community predictors may be especially helpful for smaller datasets such as the present population.

## Limitations

This study has some limitations. Our dataset is limited to claims and provides limited information about the providers. We included provider type in the model; however, having other information about the providers might be helpful for more accurate assessment of their collaboration. Our network was generated based on patient sharing relationships. Although this approach is helpful for identifying working relations among healthcare providers from claims data, it might not be a reflection of real communication between the providers. We used a multi-scale community algorithm to identify groups of healthcare providers more closely working together. Although, this algorithm addressed the resolution limit of commonly used algorithms in similar studies, the communities identified may not be the reflection of real teams. Moreover, this algorithm assigned healthcare providers to disjoint communities; however, providers may work with different groups and belong to different communities. Developing simpler algorithms which identify overlapping communities may help to generate more accurate communities from healthcare provider networks that are reflective of their real teams. Finally, we used a longitudinal analysis to study impact of provider’s network and community centrality measures on patient outcomes. Further qualitative studies might be needed to validate factors that impact the significance of network factors and the impact on patient outcomes.

## Conclusion

This study proposed a novel framework to identify network characteristics of healthcare providers involved in the care of patients with chronic conditions and the associated impact on patient outcomes. We recommend the use of this framework for health institutions interested in how team collaboration influences observed patient outcomes, and/or monitor chances in network characteristics and central providers over time. Specifically, the inclusion of community detection is recommended for analyses focused on team collaborations. For example, higher connectedness (degree) and higher access (closeness) to other providers in the community were factors that significantly reduced adverse patient outcomes in the study population. Interventions that target care managers in the network may help to improve their collaboration with other providers and the associated impact for patients.
